# Impact of mental disorders on health-related quality of life: a propensity score matched comparison

**DOI:** 10.3389/fpsyt.2025.1685750

**Published:** 2025-11-07

**Authors:** Seong-Jo Koh, Yongpyo Lee, Hye-Young Kwon

**Affiliations:** 1 Department of Social Welfare, The Catholic University of Korea, Bucheon, Republic of Korea; 2 Department of Public Health, Mokwon University, Daejeon, Republic of Korea

**Keywords:** mental disorder, schizophrenia, bipolar disorder, health-related quality of life, propensity score matching

## Abstract

**Background:**

Despite the importance of the health-related quality of life (HRQoL) in mental health, research into HRQoL among people with mental disorders remains limited in Korea.

**Aims:**

To quantify the impact of mental disorders on HRQoL in the Korean context.

**Methods:**

Propensity score matching was used to establish a case-control database. A total of 177 people with mental disorders (38.4% with schizophrenia, 21.5% with bipolar affective disorder, 20.3% with recurrent depressive disorder and 19.8% with other mental conditions) were surveyed and matched with an equal number of individuals without mental disorders from the Korean National Health and Nutrition Examination Survey. HRQoL was measured using the EuroQol-5 Dimension (EQ-5D) scale. Multivariate beta regression analysis was performed to assess the impact of mental disorders on HRQoL.

**Results:**

Individuals with mental disorders had significantly lower EQ-5D index scores (0.854, SD 0.144) than those without mental disorders (0.972, SD 0.067) (p<0.0001). According to the results of the multivariate beta regression analysis, having a mental disorder was found to significantly worsen HRQoL by 71.2%(exp(-1.244)=0.288, p<0.0001). Additionally, self-rated health was found to significantly improve HRQoL in people with mental disorders. Rating one’s health as ‘good’ was associated with a 95.1% (β=0.668, p=0.0029) increase in EQ-5D score compared to rating it as ‘poor’.

**Conclusion:**

The presence of mental disorders was significantly associated with lower HRQoL. Further in-depth studies are needed to explore HRQoL among individuals with mental disorders from a variety of perspectives, particularly within the Korean context.

## Introduction

Mental disorders have emerged as a major public health concern, affecting both personal health and social systems. Approximately 970 million people worldwide suffered from mental disorders in 2019, representing a 48.1% increase from the 654.8 million cases recorded in 1990 (1, 2). Unlike many other health conditions, mental disorders tend to have a profound impact on quality of life and functional capacity, resulting in long-term disability rather than directly causing premature death (2). Therefore, the health-related quality of life(HRQoL) of individuals with mental disorders is of great importance.

Health-related quality of life represents a multidimensional concept encompassing physical, mental, emotional, and social functioning (3, 4). It goes beyond the biological and physiological determinants of health to focus instead on the impact of health on quality of life (5, 6). In the context of mental health, HRQoL has emerged as a critical outcome measure, reflecting a transition from traditional biomedical models to holistic public health models (7). Its validity and usefulness in predicting long-term remission and recovery of mental health have been proven (8, 9). Numerous studies have investigated the factors contributing to HRQoL among individuals with mental disorders (10–17) and have consistently highlighted that these individuals reported significantly lower HRQoL scores than the general population (18–21).

For decades, Korea has had one of the highest suicide rates among OECD countries for decades (22). Given that mental ill-health increases the risk of suicide, mental health has long been a pressing issue in Korea. Recent national mental health surveys reported that the prevalence of mental disorders among adults reached 27.8% in 2021 (23) and a striking 73.6% of the population reported experiencing troubles in mental conditions such as extreme stress and depressive thoughts in 2024(up from 63.9% in 2022) (24).

Despite the increasing recognition of HRQoL in mental health, significant research gaps persist, particularly when focusing on the Korean population, few studies have examined the effect of mental disorders on HRQoL among Koreans (25–27). Studies have investigated HRQoL in people with depressive mood, panic disorder, and obsessive-compulsive disorder; however, none have focused on people with major mental disorders such as schizophrenia or bipolar disorder. Furthermore, no studies have compared HRQOL in these groups with that in healthy controls. Therefore, comprehensive investigations of HRQoL among people with mental disorders remain limited. To address this, our study aimed to investigate the impact of mental disorders on HRQoL in the Korean context, employing a propensity score-matched case-control design.

## Methods

### Study population

#### Sample population of people with mental disorders

Two hundred people with major mental disorders, aged 19 or over and residing in the community rather than in an institution, were selected using convenience sampling between July and August 2024. The sample size was allocated based on geographic and institutional distributions. The survey was conducted among individuals who frequented community-based mental health organizations, such as mental health outreach centers, psychiatric rehabilitation facilities and non-profit mental health organizations. The survey respondents reported having one of the following conditions: schizophrenia (38.4%), bipolar disorder (21.5%), recurrent depressive disorder (20.3%), or other severe mental conditions (19.8%). A semi-structured questionnaire including the EQ-5D (EuroQol 5 Dimension) was developed and administered using the KNHANES questionnaire as a reference.

#### Control population from the KNHANES

For comparison purposes, data on people without mental disorders was obtained from the KNHANES, an annual nationwide survey conducted by the Korea Disease Control and Prevention Agency(KDCA) to estimate the health awareness, behaviors, nutritional status, and HRQoL across the entire Korean population. The survey uses a stratified cluster sampling method to select a representative sample of the population. Consequently, the survey does not include separate questions about diagnosed or treated mental disorders. To establish the control group for this study, we considered the KNHANES question asking whether respondents had experienced difficulties in daily life due to depression. anxious or emotional distress. Those who answered ‘yes’ to this question were excluded to ensure the internal validity of this study. We used the most recent KNHANES HRQoL survey, conducted in 2020 (28).

#### Propensity score matching

Propensity score matching is a robust methodological approach for addressing selection bias in observational studies, particularly when examining HRQoL outcomes (29). This method enables to create comparable groups by balancing observed covariates, thereby reducing confounding factors that may influence the association between mental health conditions and HRQoL.

**Figure 1:**
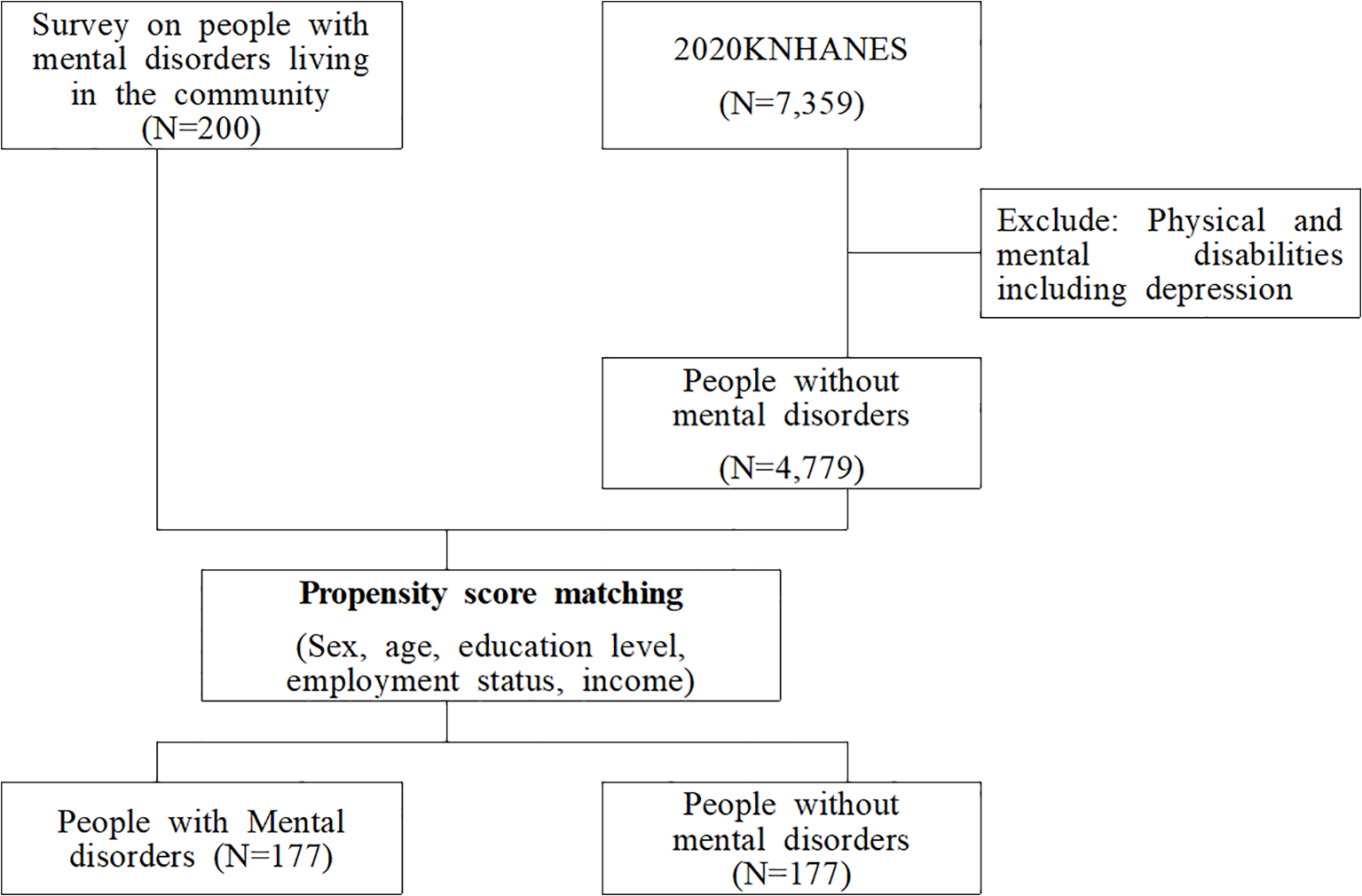
Selection process of the study population.

To identify the impact of mental disorders on HRQoL, we attempted to establish a matched population of people with and without mental disorders in a ratio of 1:1. As there is currently no nationally representative data available for directly comparing the HRQoL of these two groups, we sourced two different datasets (a sample population of people with mental disorders and a control population from the KNHANES) and used the propensity score matching (PSM) technique, which allows to minimize selection bias (30). Propensity scores were estimated using logistic regression (PROC PSMATCH). The binary variable indicating the presence of mental disorders was regressed as a function of baseline covariates. Ultimately, 177 participants were selected for each group, identical in terms of sex, age, education level, employment status and household income (see **Figure 1**).

After propensity score matching, balance diagnostics were performed to validate the matching. The matching results were evaluated using the most commonly used statistical measures: the standardized mean difference (SMD) and the variance ratio(VR). Generally, the SMD value of less than 0.25 and the VR close to 1 indicate an acceptable degree of imbalance after matching (31, 32).

### Health-related quality of life assessment

Generalized HRQoL instruments are designed to be applicable to all diseases or conditions, different medical interventions and a wide range of populations (33, 34). The EQ-5D is a generic instrument used in many countries for this purpose. It is still suitable for measuring the HRQoL of people with mental disorders (9, 35–37). The EQ-5D comprises five questions on mobility, self-care, usual activities, pain or discomfort, and psychological status, ranging 0(death) to 1(perfect health). Each item has three possible answers: 1=no problem, 2=moderate problem, and 3=severe problem. In this study, the Korean version of the EQ-5D was used to survey HRQoL in community-dwelling people with mental disorders. Responses to the individual EQ-5D dimensions were also explored.

### Covariates

Socioeconomic and health-related factors were considered as covariates. Socioeconomic factors included sex, age, education level, monthly household income, and employment status. In addition, self-rated health (poor, moderate, or good) and utilization of medical services (inpatient or outpatient) within the past 12 months were considered.

### Statistical analysis

Categorical variables were expressed as frequencies and percentages, and chi-squared tests were performed to determine the differences between the two groups. Continuous variables were expressed as means and standard deviations, and independent sample t-tests and analysis of variance (ANOVA) were performed. Where appropriate, the Mann–Whitney median test for continuous variables and Fisher’s exact test for categorical variables were performed. To analyze the differences between people with and without mental disorders, each dimension of the EQ-5D was transformed into a dichotomous variable of ‘no problem’ or ‘moderate or severe problems’.

The EQ-5D index scores, calculated based on the Korean tariff (38, 39), were analyzed using multivariate regression model with a beta logit distribution, taking into account ceiling effects and anticipated violations of normality and homoscedasticity (40). As EQ-5D scores range from 0 to 1, the bounded values were rescaled for beta regression (41). All statistical analyses were performed using the SAS software (version 9.4; SAS Institute, Inc., Cary, NC, USA).

## Results

### Basic characteristics

**Table 1** shows the basic characteristics of people with and without mental disorders before and after matching. No statistically significant differences were found between the two groups after matching in terms of sex, age, educational level, employment status, or monthly household income.

**Table 1:** Basic characteristics between the people with and without mental disorders before and after propensity score matching.

Variables		Before matching					After matching					Standardized mean difference	Variance ratio
		People with MD (N = 200)		People without MD (N = 4,779)		P-value	People with MD (N = 177)		People without MD (N = 177)		P-value		
		N	%	N	%		N	%	N	%			
Sex	female	103	51.5	2,570	53.6	NS	88	49.7	92	52.0	NS	-0.045	1.002
	male	97	48.5	2,229	46.5		89	50.3	85	48.0			
Age, Mean (SD)		40.3	13.4	50.5	16.9	<0.0001	39.7	12.8	38.9	15.4	NS	0.051	0.690
Age group	19-34	87	43.5	994	20.7	<0.0001	77	43.5	82	46.3	NS		
	35-59	97	48.5	2,168	45.2		88	49.7	72	40.7			
	60 and over	16	8	1,637	34.1		12	6.8	23	13.0			
Education level	High school or less	110	55.0	2,873	59.9	NS	96	54.2	79	44.6	NS	0.194	1.004
	Bachelor or higher	90	45.0	1,920	40.1		81	45.8	98	55.4			
Employment status	Yes	89	44.5	3,032	63.3	<0.0001	84	47.5	85	48.0	NS	0.012	0.999
	No	111	55.5	1,761	36.7		93	52.5	92	52.0			
Household Income per month (1,000 Korean Won), Mean (SD)		2,996	2,663	4,894	3,347	<0.0001	3,007	2,667	3,194	2,223	NS	-0.062	1.440

MD, Mental disorders including schizophrenia, bipolar disorder, recurrent depressive disorder and others 1USD = 1,434.42 Korean Won (as of December 2024).

A total of 354 people were matched: of them, 177 had a mental disorder and 177 did not. Among the group with mental disorders, the proportion of men and women was equal, with an average age of 39.7 (SD 12.5). Of this group, 45.8% had a college degree or higher and 47.5% were in employment with an average monthly income of 3,007 (SD 2,667) thousand Korean won (KRW). In the group without mental disorders, 52% were male, with an average age of 38.9 (SD 15.4). 55.4% of this group had a college degree or higher and 48.0% were employed, earning an average monthly income of 3,194 (SD 2,223) thousand KRW. However, there were no statistical differences between the two groups in terms of any of the variables, as the two groups were matched by their socio-demographic characteristics. The matching was balanced because the SMDs for these variables were less than 0.25 and their VRs were close to 1.

### Health status

**Table 2** illustrates the approximate differences in health status between the two groups, including EQ-5D scores, self-rated health, and inpatient or outpatient service use within the past 12 months. People with mental disorders had significantly lower EQ-5D index scores (0.854, SD 0.144 vs. 0.972 SD 0.067, p<0.0001), a higher rate of “poor” health (31.6% vs. 15.8%, p=0.0006), a higher rate of hospitalizations (20.3% vs. 4.5%, p<0.0001) and a significantly higher rate of outpatient service use in the past 12 months (94.3% vs. 17.5%, p<0.0001) than the control group.

**Table 2:** Differences in health status between the two groups in comparison(unadjusted).

Variables		People with MD (N = 177)		People without MD (N = 177)		P-value
		N	%	N	%	
EQ-5D Index scores (Mean, SD)		0.854	0.144	0.972	0.067	<0.0001
Self-rated health						
	Poor	56	31.6	28	15.8	0.0006
	Moderate	85	48.0	91	51.4	
	Good	36	20.3	58	32.8	
Medical service use within the previous 12 months						
Hospitalizations	Yes	36	20.3	8	4.5	<0.0001
	No	141	79.7	169	95.5	
Outpatient	Yes	164	94.2	31	17.5	<0.0001
	No	10	5.8	146	82.5	

MD, Mental disorders; EQ-5D, EuroQol-5 dimension.

### EQ-5D

**Figure 2:**
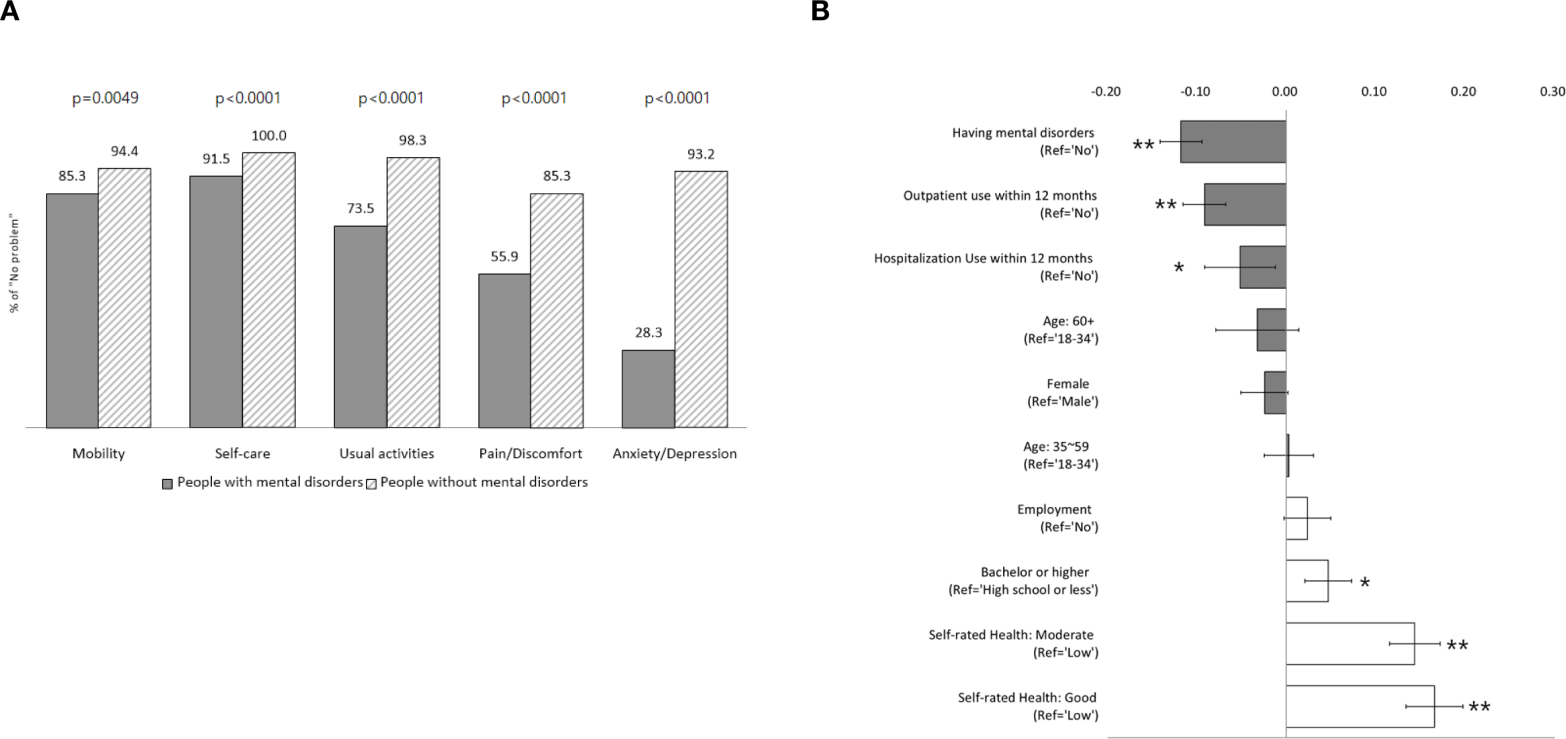
Comparison of EQ-5D between people with and without mental disorders. **(A)** Differences in the distribution of each dimension of EQ-5D between People with and without mental disorders. **(B)** Mean differences of EQ-5D scores between people with and without mental disorders (unadjusted). *p<0.05, **p<0.001.

**Figure 2A** shows the five EQ-5D dimensions converted into dichotomous variables and comparatively analyzed between the two groups. People with mental disorders tended to report a significantly lower fewer “no problem” than the comparison group for all dimensions: “mobility” (85.3% vs. 94.4%, p=0.0049), “self-care” (91.5% vs. 100%, p<0.0001), “usual activities” (73.5% vs. 98.3%, p<0.0001), “pain/discomfort” (55.9% vs. 85.3%, p<0.0001), and “anxiety/depression” (28.3% vs. 93.2%, p<0.0001). More specifically, 44.1% of people with mental disorders experienced impairment in the “pain/discomfort” dimension, and 71.7% reported anxiety or depression.

**Figure 2B** illustrates the unadjusted mean difference in the EQ-5D scores across each variable. The mean EQ-5D scores differed significantly depending on whether an individual had a mental disorder, as well as according to their education level, employment status, medical service utilization in the past 12 months, and self-rated health. On average, people with mental disorders had EQ-5D scores that were 0.118 points lower than those without mental disorders (p<0.0001). Those who had used outpatient services within the past 12 months had significantly lower EQ-5D scores (by 0.092) than those who had not (p<0.0001). Similarly, those hospitalized within the previous 12 months had an EQ-5D score that was 0.052 lower than those who had not (p=0.011). Higher education levels were associated with significantly higher EQ-5D scores (p=0.001). Self-rated health was also a significant factor influencing HRQoL. Those who rated their health as ‘good’ or ‘moderate’ had EQ-5D scores that were 0.166 and 0.144 higher, respectively, than those who rated their health as “poor” (p<0.0001).

### Multivariate beta regression

**Table 3** shows the results of the multivariate beta regression analysis investigating the factors influencing EQ-5D scores. Since our study population was matched for sex, age, education level, employment status, and household income between those with and without mental disorders, only two variables–mental disorders and self-rated health–were statistically significant. After adjusting for other variables, individuals with mental disorders had 0.288 times(-71.2%) (exp(−1.244)=0.288, p<0.0001) lower HRQoL scores than the group without mental disorders. Regarding self-rated health, HRQoL was 76.5% (p=0.0072) higher for ‘moderate’ health and 95.1% (p=0.0029) higher for ‘good’ health compared to ‘poor’ health. The interaction between mental disorders and self-rated health was also examined; however, the results were not statistically significant (p = 0.0678). Mental disorders consistently showed a significantly negative association with HRQoL across the self-rated health statuses (see **Supplementary Table 1**). Hospitalization and outpatient service use within the past 12 months were not significantly associated with HRQoL.

**Table 3:** Results of multivariate beta regression for EQ-5D index scores.

Variables		Estimate	Standard error	P-value
Intercept		2.713	0.275	<0.0001
Mental disorders	Yes	-1.244	0.238	<0.0001
	(ref=No)	0		
Sex	Female	-0.074	0.101	NS
	(ref=male)	0		
Age		-0.004	0.004	NS
Education level	Bachelor or higher	0.153	0.103	NS
	(ref=High school or less)	0		
Employment	Yes	0.034	0.104	NS
	(ref=No)	0		
Household Income		-0.0002	0.0002	NS
Hospitalization within the past 12 months	Yes	-0.002	0.150	NS
	(ref=No)	0		
Outpatient Service use within the past 12 months	Yes	-0.087	0.175	NS
	(ref=No)	0		
Self-rated health	Moderate	0.568	0.210	0.0072
	Good	0.668	0.223	0.0029
	(ref=Poor)	0		

The interaction term of having mental disorders and self-rated health had a marginally significant effect on EQ-5D scores (p=0.0678).

## Discussion

This study sought to evaluate the impact of mental disorders on HRQoL by comparing EQ-5D scores between individuals with and without mental disorders. A total of 177 people with mental disorders were recruited to complete the EQ-5D survey. As a control group, participants from the 2020 KNHANES were matched to the survey group using propensity scores based on sex, age, education level, employment status, and household income. Beta regression analysis was conducted to quantify the impact of having a mental disorder on HRQoL.

The results showed that individuals with mental disorders had significantly lower EQ-5D scores compared to those without mental disorders (0.854 SD 0.144 vs. 0.972 SD 0.067, p<0.0001). Specifically, the beta regression analysis revealed that individuals with mental disorders had 71.2% lower HRQoL than those without. This finding aligns with previous research (18–20). For instance, a Swedish study found that individuals with mental disorders had substantially lower HRQoL compared to the general population (EQ-5D scores 0.727 vs. 0.812) (19). Similarly, a Finnish study found that individuals with schizophrenia had significantly lower EQ-5D scores (0.715, SD 0.041) than those without psychosis (0.838, SD 0.003) (20). Other measure such as SF-6 (Short Form 6 Health Survey), SF-12, and WHOQOL-BREF (World Health Organization Quality of Life-Brief Version) have also been used to measure HRQoL among people with mental disorders, and similar results have been reported (18, 21, 42, 43). For example, a French study using the SF-6 showed significantly lower HRQOL scores among individuals with mental disorders than the general population (0.683, SD 0.121 vs. 0.766, SD 0.137) (21). This is crucially employed in health economics to estimate the quality-adjusted life years(QALY) of mental disorders.

We also found that individuals with mental disorders experienced worse health in all EQ-5D dimensions compared to those without mental disorders. The largest differences were observed in ‘anxiety/depression’ (28.3% vs. 93.2%, p<0.0001). ‘Usual activities’ showed the next largest gap (73.5% vs. 98.3%, p<0.0001), followed by ‘pain/discomfort’ (55.9% vs. 85.3%, p<0.0001). These findings align with a previous study that reported worse HRQoL in all dimensions, except ‘pain/discomfort’, among mental disorder patients (19). Our sample reported a higher prevalence of ‘pain/discomfort’, suggesting that individuals with mental disorders may experience both physical and psychological suffering. This is not consistent with the previous findings[19].Individuals with mental disorders may have a high risk of metabolic syndrome due to an unhealthy lifestyle caused by depression or anxiety, as well as the adverse effects of antipsychotics. This can potentially lead to chronic pain and physical discomfort (44, 45). Conversely, they may also experience impaired pain processing (46), the analgesic effects of antipsychotics (47), or reduced pain sensitivity (48), which may result in under-reporting of pain. Therefore, to better understand ‘pain/discomfort’ in individuals with mental disorders, it is necessary to consider the complex interplay between pain sensitivity and psychological and therapeutic factors (49).

Notably, this study identified a significant positive association between self-rated health and HRQoL. Participants who rated their health as ‘moderate’ demonstrated 76.5% higher HRQoL scores (p=0.0072). Those who rated their health as ‘good’ had 95.1% higher HRQoL scores (p=0.0029), compared to the ‘poor’ group’. Within the control group, 32.8% rated their health as ‘good’, while only 20.3% of those with mental disorders did so. These findings emphasize the critical need for targeted interventions to improve self-rated health among individuals with mental disorders. This positive association between self-rated health and HRQoL is supported by previous findings. For instance, Paul et al. (2023) reported that self-rated health accounted for 43% of the variation in HRQoL, recommending its use as a surrogate measure in primary care (50). Other studies showed that self-rated health can be improved through social activities, such as employment, social participation, and networking (51, 52). Therefore, enhancing social integration—by encouraging active participation, building networks, and fostering a sense of belonging—may improve self-rated health in this group. In line with this, Defar et al. (2023) found that HRQoL among individuals with mental disorders was significantly associated with social support, employment, and functional disability (14). Therefore, a multidisciplinary approach that considers both social and clinical factors is essential to improve HRQoL in this population (13, 14, 17).

This study has several limitations. First, we combined the two data sources to compare HRQoL between individuals with and without mental disorders. Due to lack of a reliable health survey specifically for individuals with mental disorders comparable to the KNHANES, we conducted a separate survey for this group. We then used matched controls from the KNHANES. Despite the heterogeneity between the two datasets, we tried to provide insights into HRQoL in Koreans with mental disorders – a topic that has received little research attention. Second, when selecting healthy controls from the KNHANES, we excluded respondents who reported experiencing difficulties in daily life due to depressive, anxious symptoms or emotional distress. This resulted in the strict exclusion of individuals with temporary or minimal mental conditions. This might lead to an overestimation of EQ-5D scores among health controls. Third, sampling bias may be present in this study. Unlike the KNHANES, our survey used non-probabilistic sampling and focused on individuals utilizing community mental health welfare services, who may have relatively mild to moderate conditions compared to those in institutional care. Thus, the findings may not represent all people with mental disorders. Actual EQ-5D scores for this population may be lower than the observed value of 0.854 (SD 0.144). Lastly, omitted variable bias may have affected the results. We could not adjust for certain covariates, such as comorbidities, lifestyle, morbidity duration, or cognitive function due to data limitations. Therefore, our findings should be interpreted with caution because of the limited generalizability.

Despite these limitations, this study is the first to quantify the negative impact of mental disorders on HRQoL in Korea using a propensity score matching. A key strength of this study is its focus on HRQoL among people with mental disorders, an area that has largely been overlooked in Korean research. The findings highlight the need for a nationwide health survey incorporating EQ-5D questionnaires, as well as implementing community-based strategies such as social integration initiatives or job support services, to enhance self-rated health among individuals with mental disorders.

## Conclusion

The presence of mental disorders was significantly associated with lower HRQoL. Further in-depth studies are needed to explore HRQoL among individuals with mental disorders from a variety of perspectives, particularly within the Korean context.

## Data Availability

The raw data supporting the conclusions of this article will be made available by the authors, without undue reservation.

## Supplementary material

Supplementary Table 1 Results of multivariate beta regression stratified by Self-rated health.

## References

[1] WHO . Mental disorders (2022). Available online at: https://www.who.int/news-room/fact-sheets/detail/mental-disorders (Accessed March 10, 2025).

[2] GBD . 2019 Mental Disorders Collaborators. Global, regional, and national burden of 12 mental disorders in 204 countries and territories, 1990–2019: a systematic analysis for the Global Burden of Disease Study 2019. Lancet Psychiatry. (2022) 9:137–50. doi: 10.1016/S2215-0366(21)00395-3, PMID: 35026139 PMC8776563

[3] Karimi M Brazier J . Health, health-related quality of life, and quality of life: what is the difference? Pharmacoeconomics. (2016) 34:645–9. doi: 10.1007/s40273-016-0389-9, PMID: 26892973

[4] Testa MA Simonson DC . Assessment of quality-of-life outcomes. New Engl J Med. (1996) 334:835–40. doi: 10.1056/NEJM199603283341306, PMID: 8596551

[5] Guyatt GH Ferrans CE Halyard MY Revicki DA Symonds TL Varricchio CG . Exploration of the value of health-related quality-of-life information from clinical research and into clinical practice. Mayo Clinic Proc. (2007) 82:1229–39. doi: 10.4065/82.10.1229, PMID: 17908529

[6] Kobau R Sniezek J Zack MM Lucas RE Burns A . Well-being assessment: an evaluation of well-being scales for public health and population estimates of well-being among US adults. Appl Psychology: Health Well Being. (2010) 2:272–97. doi: 10.1111/j.1758-0854.2010.01035.x

[7] Revicki DA Kleinman L Cella D . A history of health-related quality of life outcomes in psychiatry. Dialogues Clin Neurosci. (2014) 16:127–35. doi: 10.31887/DCNS.2014.16.2/drevicki, PMID: 25152652 PMC4140507

[8] Karow A WL Schöttle D Schäfer I Lambert M . The assessment of quality of life in clinical practice in patients with schizophrenia. Dialogues Clin Neurosci. (2014) 16:185–95. doi: 10.31887/DCNS.2014.16.2/akarow, PMID: 25152657 PMC4140512

[9] Mulhern B Mukuria C Barkham M Knapp M Byford S Soeteman D . Using generic preference-based measures in mental health: Psychometric validity of the EQ-5D and SF-6D. Br J Psychiatry. (2014) 205:236–43. doi: 10.1192/bjp.bp.112.122283, PMID: 24855127

[10] Alonso J Croudace T Brown J Gasquet I Knapp MRJ Suárez D . Health-related quality of life (HRQL) and continuous antipsychotic treatment: 3-year results from the schizophrenia health outcomes (SOHO) study. Value Health. (2009) 12:536–43. doi: 10.1111/j.1524-4733.2008.00495.x, PMID: 19900255

[11] Domenech C Altamura C Bernasconi C Corral R Elkis H Evans J . Health-related quality of life in outpatients with schizophrenia: factors that determine changes over time. Soc Psychiatry Epidemiol. (2018) 53:239–48. doi: 10.1007/s00127-018-1483-4, PMID: 29340780

[12] Lim MWZ Lee J . Determinants of health-related quality of life in schizophrenia: beyond the medical model. Front Psychiatry. (2018) 9:712. doi: 10.3389/fpsyt.2018.00712, PMID: 30618882 PMC6305274

[13] Arraras JI Ibañez B Basterra I Pereda N Martin M Iribarren S . Determinants of Quality of Life in Spanish outpatients with schizophrenia spectrum disorders. Eur J Psychiatry. (2018) 32:113–21. doi: 10.1016/j.ejpsy.2017.11.001

[14] Defar S Abraham Y Reta Y Deribe B Jisso M Yeheyis T . Health related quality of life among people with mental illness: The role of socio-clinical characteristics and level of functional disability. Front Public Health. (2023) 11:1134032. doi: 10.3389/fpubh.2023.1134032, PMID: 36875411 PMC9978447

[15] Oyama H Oda K Matsuo R . Factors associated with health-related quality of life in long-stay inpatients with chronic schizophrenia. PCN Rep. (2022) 1:e42. doi: 10.1002/pcn5.42, PMID: 38868685 PMC11114288

[16] Alemu WG Due C Muir-Cochrane E Mwanri L Azale T Ziersch A . Quality of life among people living with mental illness and predictors in Africa: a systematic review and meta-analysis. Qual Life Res. (2024) 33:1191–209. doi: 10.1007/s11136-023-03525-8, PMID: 37906348 PMC11045618

[17] Havnen A Lindberg MS Lundqvist J Brattmyr M Hjemdal O Solem S . Health-related quality of life in psychiatric outpatients: a cross-sectional study of associations with symptoms, diagnoses, and employment status. Qual Life Res. (2024) 33:3093–3105. doi: 10.1007/s11136-024-03748-3, PMID: 39110377 PMC11541330

[18] Dong M Lu L Zhang L Zhang YS Ng CH Ungvari GS . Quality of life in schizophrenia: A meta-analysis of comparative studies. Psychiatr Quarterly. (2019) 90:519–32. doi: 10.1007/s11126-019-09633-4, PMID: 31119453

[19] Foldemo A Wärdig R Bachrach-Lindström M Edman G Holmberg T Lindström T . Health-related quality of life and metabolic risk in patients with psychosis. Schizophr Res. (2014) 152:295–9. doi: 10.1016/j.schres.2013.11.029, PMID: 24355528

[20] Saarni SI Viertiö S Perälä J Koskinen S Lönnqvist J Suvisaari J . Quality of life of people with schizophrenia, bipolar disorder and other psychotic disorders. Br J Psychiatry. (2010) 197:386–94. doi: 10.1192/bjp.bp.109.076489, PMID: 21037216

[21] Prigent A Auraaen A Kamendje-Tchokobou B Durand-Zaleski I Chevreul K . Health-related quality of life and utility scores in people with mental disorders: A comparison with the non-mentally ill general population. Int J Environ Res Public Health. (2014) 11:2804–17. doi: 10.3390/ijerph110302804, PMID: 24608903 PMC3987005

[22] OECD . Society at a glance 2024: OECD social indicators. Paris: OECD Publishing (2024). doi: 10.1787/918d8db3-en

[23] Rim SJ Hahm BJ Seong SJ Park JE Chang SM Kim BS . Prevalence of mental disorders and associated factors in korean adults: national mental health survey of korea 2021. Psychiatry Invest. (2023) 20:262–72. doi: 10.30773/pi.2022.0307, PMID: 36990670 PMC10064208

[24] Ministry of Health and Welfare . The National Mental Health Knowledge and Attitude Survey 2024 [press release]. (2024). Available from: https://www.mohw.go.kr/board.es?mid=a10503010100&bid=0027&act=view&list_no=1482175&tag=&nPage=1

[25] Cho Y Lee JK Kim DH Park JH Choi M Kim HJ . Factors associated with quality of life in patients with depression: A nationwide population-based study. PloS One. (2019) 14:e0219455. doi: 10.1371/journal.pone.0219455, PMID: 31295291 PMC6623963

[26] Son MH Byun KR Choi BH Woo JM . Assessment of health-related quality of life among patients with panic disorder using euroQol in korea. Anxiety Mood. (2012) 8:9–15. doi: 10.22857/kjbp.2017.24.3.004

[27] Kim SN Moon W Han JW . Association between quality of life and symptom severity in obsessive-compulsive disorder patients using EQ-5D. Korean J Biol Psychiatry. (2017) 24:129–33. doi: 10.0000/kjbp.2017.24.3.129

[28] Korean Center for Disease Control and Prevention (KCDC) . The Eighth Korea National Health and Nutrition Examination Survey (KNHANES VIII) 2019–2021: User Guide. Seoul: Korean Center for Disease Control and Prevention.

[29] Austin PC . An introduction to propensity score methods for reducing the effects of confounding in observational studies. Multivariate Behav Res. (2011) 46:399–424. doi: 10.1080/00273171.2011.568786, PMID: 21818162 PMC3144483

[30] D’Agostino RB . Propensity score methods for bias reduction in the comparison of a treatment to a non-randomized control group. Stat Med. (1998) 17:2265–81. doi: 10.1002/(SICI)1097-0258(19981015)17:19<2265::AID-SIM918>3.0.CO;2-B, PMID: 9802183

[31] Zhang Z Kim HJ Lonjon G Zhu Y written on behalf of AME Big-Data Clinical Trial Collaborative Group . Balance diagnostics after propensity score matching. Ann Trans Med. (2019) 7:16. doi: 10.21037/atm.2018.12.10, PMID: 30788363 PMC6351359

[32] Stuart EA . Matching methods for causal inference: A review and a look forward. Stat Sci. (2010) 25:1. doi: 10.1214/09-STS313, PMID: 20871802 PMC2943670

[33] EuroQol Group . EuroQol--a new facility for the measurement of health-related quality of life. Health Policy. (1990) 16:199–208. doi: 10.1016/0168-8510(90)90421-9, PMID: 10109801

[34] Patrick DL Deyo RA . Generic and disease-specific measures in assessing health status and quality of life. Med Care. (1989) 27:S217–S32. doi: 10.1097/00005650-198903001-00018, PMID: 2646490

[35] Stochl J Croudace T Perez J Birchwood M Lester H Marshall M . Usefulness of EQ-5D for evaluation of health-related quality of life in young adults with first-episode psychosis. Qual Life Res. (2013) 22:1055–63. doi: 10.1007/s11136-012-0222-7, PMID: 22706728

[36] Pitkanen A Valimaki M Endicott J Katajisto J Luukkaala T Koivunen M . Assessing quality of life in patients with schizophrenia in an acute psychiatric setting: reliability, validity and feasibility of the EQ-5D and the Q-LES-Q. Nordic J Psychiatry. (2012) 66:19–25. doi: 10.3109/08039488.2011.593099, PMID: 21770824

[37] Subramaniam M Abdin E Poon LY Vaingankar JA Lee H Chong SA . EQ-5D as a measure of program outcome: Results from the Singapore early psychosis intervention program. Psychiatry Res. (2014) 215:46–51. doi: 10.1016/j.psychres.2013.10.002, PMID: 24210666

[38] Lee YK Nam HS Chuang LH Kim KY Yang HK Kwon IS . South korean time trade-off values for EQ-5D health states: modeling with observed values for 101 health states. Value Health. (2009) 12:1187–93. doi: 10.1111/j.1524-4733.2009.00579.x, PMID: 19659703

[39] Jo MW Yun SC Lee SI . Estimating quality weights for EQ-5D health states with the time trade-off method in South Korea. Value Health. (2008) 11:1186–9. doi: 10.1111/j.1524-4733.2008.00348.x, PMID: 18489498

[40] Hunger M Döring A Holle R . Longitudinal beta regression models for analyzing health-related quality of life scores over time. BMC Med Res Methodology. (2012) 12:1–12. doi: 10.1186/1471-2288-12-144, PMID: 22984825 PMC3528618

[41] Verkuilen J Smithson M . Mixed and mixture regression models for continuous bounded responses using the beta distribution. J Educ Behav Stat. (2016) 37:82–113. doi: 10.3102/1076998610396895

[42] Hofer A Mizuno Y Wartelsteiner F Wolfgang Fleischhacker W Frajo-Apor B Kemmler G . Quality of life in schizophrenia and bipolar disorder: The impact of symptomatic remission and resilience. Eur Psychiatry. (2017) 46:42–7. doi: 10.1016/j.eurpsy.2017.08.005, PMID: 28992535

[43] Chang J Cho J Medina M Falcon S Soto-Ruiz P Shin DY . Factors associated with Health-Related Quality of Life in Hispanic population with mental disorders using medical expenditure panel survey 2013-2017. J Hospial Administration. (2021) 10:1. doi: 10.5430/jha.v10n3p1

[44] Kato T Baba K Guo W Chen Y Nosaka T . Impact of bipolar disorder on health-related quality of life and work productivity: estimates from the national health and wellness survey in Japan. J Affect Disord. (2021) 295:203–14. doi: 10.1016/j.jad.2021.07.104, PMID: 34479128

[45] Saccaro LF Aimo A Panichella G Sentissi O . Shared and unique characteristics of metabolic syndrome in psychotic disorders: a review. Front Psychiatry. (2024) 15:1343427. doi: 10.3389/fpsyt.2024.1343427, PMID: 38501085 PMC10944869

[46] Zhou L Bi Y Liang M Kong Y Tu Y Zhang X . A modality-specific dysfunction of pain processing in schizophrenia. Hum Brain Mapping. (2020) 41:1738–53. doi: 10.1002/hbm.24906, PMID: 31868305 PMC7267942

[47] Jimenez XF Sundararajan T Covington EC . A systematic review of atypical antipsychotics in chronic pain management: olanzapine demonstrates potential in central sensitization, fibromyalgia, and headache/migraine. Clin J pain. (2018) 34:585–91. doi: 10.1097/AJP.0000000000000567, PMID: 29077621

[48] González-Rodríguez A Labad J Seeman MV . Pain sensitivity in schizophrenia spectrum disorders: A narrative review of recent work. Psychiatry Int. (2021) 2:48–58. doi: 10.3390/psychiatryint2010004

[49] Li AL Peng YB . Comorbidity of depression and pain: a review of shared contributing mechanisms. J Neurol Neuromedicine. (2014) 2:112. doi: 10.29245/2572.942X/2017/3.1116

[50] Paul N Cittadino J Weiss B Krampe H Denke C Spies CD . Subjective ratings of mental and physical health correlate with EQ-5D-5L index values in survivors of critical illness: A construct validity study. Crit Care Med. (2023) 51:365–75. doi: 10.1097/CCM.0000000000005742, PMID: 36606801 PMC9936981

[51] Nieminen T Martelin T Koskinen S Aro H Alanen E Hyyppä MT . Social capital as a determinant of self-rated health and psychological well-being. Int J Public Health. (2010) 55:531–42. doi: 10.1007/s00038-010-0138-3, PMID: 20361226

[52] Nagao-Sato S Akamatsu R Karasawa M Tamaura Y Fujiwara K Nishimura K . Associations between patterns of participation in community activities and social support, self-efficacy, self-rated health among community-dwelling older adults. J Psychiatr Res. (2023) 157:82–7. doi: 10.1016/j.jpsychires.2022.11.023, PMID: 36455377

