# Supplementary material for: Impact of mental disorders on health-related quality of life: a propensity score matched comparison
Source: Front Psychiatry. 2025 Nov 7;16:1685750. doi: 10.3389/fpsyt.2025.1685750 (PMC12634661; doi:10.3389/fpsyt.2025.1685750)
Supplement: Supplementary Table 1 — Results of multivariate beta regression stratified by Self-rated health. [file Table1.docx]

Supplementary Table 1. Results of Multivariate Beta regression stratified by Self-rated Health

|  | | Good(N=84) | | | Moderate(N=176) | | | Low(N=91) | | |
| --- | --- | --- | --- | --- | --- | --- | --- | --- | --- | --- |
| Variables | | Estimate | S.E | P-value | Estimate | S.E | P-value | Estimate | S.E | P-value |
|  |  |  |  |  |  |  |  |  |  |  |
| Intercept | | 2.732 | 0.458 | <0.0001 | 3.459 | 0.346 | <.0001 | 3.098 | 0.431 | <.0001 |
| ***Mental disorders*** | ***Yes*** | ***-1.342*** | ***0.364*** | ***0.0004*** | ***-0.628*** | ***0.263*** | ***0.018*** | ***-0.829*** | ***0.353*** | ***0.0214*** |
|  | ***(ref=No)*** | ***0*** | ***.*** | ***.*** | ***0*** | ***.*** | ***.*** | ***0*** | ***.*** | ***.*** |
| Sex | Female | -0.050 | 0.214 | NS | -0.097 | 0.143 | NS | -0.012 | 0.220 | NS |
|  | (ref=male) | 0 | . | . | 0 | . | . | 0 | . | . |
| Age |  | -0.014 | 0.007 | NS | 0.004 | 0.006 | NS | 0.009 | 0.009 | NS |
| Education level | Bachelor or higher | 0.227 | 0.218 | NS | -0.021 | 0.146 | NS | 0.319 | 0.216 | NS |
|  | (ref=High school or less) | 0 | . | . | 0 | . | . | 0 | . | . |
| Employment | Yes | 0.174 | 0.229 | NS | -0.044 | 0.146 | NS | 0.040 | 0.208 | NS |
|  | (ref=No) | 0 | . | . | 0 | . | . | 0 | . | . |
| Household Income | | -0.0002 | 0.001 | NS | -0.0004 | 0.0003 | NS | 0.000 | 0.000 | NS |
| Hospitalization  within the past 12 months | Yes | -0.052 | 0.295 | NS | 0.155 | 0.215 | NS | -0.386 | 0.320 | NS |
|  | (ref=No) | 0 | . | . | 0 | . | . | 0 | . | . |
| Outpatient Service use within the past 12 months | Yes | 0.254 | 0.388 | NS | -0.525 | 0.258 | 0.043 | 0.065 | 0.330 | NS |
|  | (ref=No) | 0 | . | . | 0 | . | . | 0 | . | . |
